# First Report of *cfr*-Carrying Plasmids in the Pandemic Sequence Type 22 Methicillin-Resistant Staphylococcus aureus Staphylococcal Cassette Chromosome *mec* Type IV Clone

**DOI:** 10.1128/AAC.02949-15

**Published:** 2016-04-22

**Authors:** Anna C. Shore, Alexandros Lazaris, Peter M. Kinnevey, Orla M. Brennan, Gráinne I. Brennan, Brian O'Connell, Andrea T. Feßler, Stefan Schwarz, David C. Coleman

**Affiliations:** a Microbiology Research Unit, Dublin Dental University Hospital, University of Dublin, Trinity College Dublin, Dublin, Ireland; b National MRSA Reference Laboratory, St. James's Hospital, Dublin, Ireland; c Department of Clinical Microbiology, School of Medicine, Trinity College Dublin, St. James's Hospital, Dublin, Ireland; d Institute of Farm Animal Genetics, Friedrich Loeffler Institut, Neustadt-Mariensee, Germany

## Abstract

Linezolid is often the drug of last resort for serious methicillin-resistant Staphylococcus aureus (MRSA) infections. Linezolid resistance is mediated by mutations in 23S rRNA and genes for ribosomal proteins; *cfr*, encoding phenicol, lincosamide, oxazolidinone, pleuromutilin, and streptogramin A (PhLOPS_A_) resistance; its homologue *cfr*(B); or *optrA*, conferring oxazolidinone and phenicol resistance. Linezolid resistance is rare in S. aureus, and *cfr* is even rarer. This study investigated the clonality and linezolid resistance mechanisms of two MRSA isolates from patients in separate Irish hospitals. Isolates were subjected to *cfr* PCR, PhLOPS_A_ susceptibility testing, 23S rRNA PCR and sequencing, DNA microarray profiling, *spa* typing, pulsed-field gel electrophoresis (PFGE), plasmid curing, and conjugative transfer. Whole-genome sequencing was used for single-nucleotide variant (SNV) analysis, multilocus sequence typing, L protein mutation identification, *cfr* plasmid sequence analysis, and *optrA* and *cfr*(B) detection. Isolates M12/0145 and M13/0401 exhibited linezolid MICs of 64 and 16 mg/liter, respectively, and harbored identical 23S rRNA and L22 mutations, but M12/0145 exhibited the mutation in 2/6 23S rRNA alleles, compared to 1/5 in M13/0401. Both isolates were sequence type 22 MRSA staphylococcal cassette chromosome *mec* type IV (ST22-MRSA-IV)/*spa* type t032 isolates, harbored *cfr*, exhibited the PhLOPS_A_ phenotype, and lacked *optrA* and *cfr*(B). They differed by five PFGE bands and 603 SNVs. Isolate M12/0145 harbored *cfr* and *fexA* on a 41-kb conjugative pSCFS3-type plasmid, whereas M13/0401 harbored *cfr* and *lsa*(B) on a novel 27-kb plasmid. This is the first report of *cfr* in the pandemic ST22-MRSA-IV clone. Different *cfr* plasmids and mutations associated with linezolid resistance in genotypically distinct ST22-MRSA-IV isolates highlight that prudent management of linezolid use is essential.

## INTRODUCTION

The oxazolidinone antimicrobial agent linezolid was first introduced into clinical practice in 2000, and it quickly became the drug of last resort to treat skin and soft tissue infections and pneumonia caused by multidrug-resistant Gram-positive cocci, including methicillin-resistant Staphylococcus aureus (MRSA). Linezolid binds to the A site of the peptidyl transferase center in the V domain of the 23S rRNA component of the 50S subunit of the bacterial ribosome (1). Binding of linezolid interferes with the correct positioning of aminoacyl tRNA on the ribosome, which prevents the formation of the initiation complex and thus inhibits the initiation of protein synthesis (1).

Resistance to linezolid is predominantly mediated by (i) mutations in the drug target site (domain V of the six 23S rRNA alleles) or in the genes encoding the 50S ribosomal proteins (L3, L4, and L22) that have been speculated to result in the impairment of linezolid binding and/or (ii) acquisition of the transferable linezolid resistance gene *cfr* (2–4). The *cfr* gene encodes a methyltransferase that catalyzes the posttranscriptional methylation of adenosine at nucleotide position 2503 (Escherichia coli numbering) in 23S rRNA, thus interfering with the binding of linezolid to its target (4, 5). However, due to overlapping binding sites, *cfr* methylation also affects the binding of four other classes of antimicrobial agents and results in the multiresistance PhLOPS_A_ phenotype, i.e., resistance to phenicols, lincosamides, oxazolidinones, pleuromutilins and streptogramin A compounds (6). Recently, a novel plasmid-located ABC transporter gene, *optrA*, conferring resistance to linezolid and phenicols, and a *cfr* homologue, *cfr*(B), have also been identified (7–9).

The *cfr* gene was first reported in a bovine Staphylococcus sciuri isolate in 1997 and was subsequently found in many different staphylococcal species, including methicillin-susceptible S. aureus (MSSA), MRSA, and coagulase-negative and coagulase-variable (Staphylococcus hyicus) staphylococci, as well as in Bacillus, Enterococcus, Streptococcus, Macrococcus, Jeotgalicoccus, Proteus, and Escherichia species (10–12). It has been detected in isolates from humans, livestock, meat products, and the environment and has been identified on a variety of plasmids, although chromosomal locations have also been reported (10, 13). In some instances, different bacterial species as well as a variety of animal and human hosts have been found to harbor similar *cfr* plasmids or genetic environments, highlighting the ability of *cfr* to spread (10). Specific insertion sequences (ISs) have been shown to play a role in *cfr* mobility and integration into different plasmid types, and *cfr* is often colocated with other resistance determinants, allowing for the coselection of *cfr* (10).

Linezolid resistance remains relatively rare among S. aureus isolates, and *cfr* is even more so (14–16). The earliest reported *cfr*-mediated linezolid-resistant S. aureus isolates were two sequence type 5 (ST5) MRSA isolates recovered in 2005 from two patients in hospitals in Colombia and Indianapolis, IN, respectively (17, 18). The *cfr* gene was subsequently reported in a small number of sporadically occurring S. aureus isolates, predominantly MRSA, from both animals and humans, belonging to a range of genotypes, including the multilocus sequence type (MLST) clonal complex 5 (CC5) (STs 627, 228, 5, 125, and 1788), CC6/ST6, CC8/ST8 (ST8 MRSA staphylococcal cassette chromosome *mec* [SCC*mec*] type IV [ST8-MRSA-IV]/USA300), CC9 (STs 9 and 63), and CC398/ST398 as well as in association with an outbreak of an unspecified MRSA clone in a Spanish hospital in 2008 (17, 19–32). Although two studies localized *cfr* to the S. aureus chromosome (one within the SCC*mec* type IVb J1 region [23] and one within 23S rRNA allele 4 [18, 33]), it has predominantly been reported to be located on a diverse range of plasmids (10).

In Ireland, only one *cfr*-positive MRSA isolate has been reported to date (USA300/ST8-MRSA-IVa), in which *cfr* was located on a novel plasmid (pSCFS7) together with a second phenicol resistance gene, *fexA*, via the integration of *cfr* into the *fexA*-carrying transposon Tn*558* (20). Recently, *cfr* was also detected in methicillin-resistant Staphylococcus epidermidis (MRSE) clinical isolates from Ireland, although the possible plasmid location of *cfr* in these isolates was not reported (34, 35).

ST22-MRSA-IV is a pandemic MRSA clone that is endemic in hospitals in Ireland and the United Kingdom and predominates among nosocomial MRSA isolates in several other European countries, Asia, and Australia (36–41). It has also been reported sporadically in the United States and South America (42, 43). Although mutational resistance to linezolid has been reported in ST22-MRSA-IV isolates, *cfr* has not been reported (44). During 2012 and 2013, two epidemiologically unrelated linezolid-resistant MRSA isolates were recovered from two patients in two separate Irish hospitals and were submitted to the Irish National MRSA Reference Laboratory. The purpose of this study was to investigate the genetic basis of linezolid resistance and the genetic relatedness of these isolates. This study reports the first identification of *cfr* in association with two distinct *cfr* plasmids in two genetically distinct ST22-MRSA-IV isolates.

## MATERIALS AND METHODS

### Bacterial isolates.

Two linezolid-resistant MRSA isolates recovered from patients in two separate Irish hospitals ∼250 km apart, one in 2012 in Cork (M12/0145) and the other in 2013 in Dublin (M13/0401), were investigated. Isolate M12/0145 was recovered from a sputum sample, and the patient had previously been treated with linezolid. Isolate M13/0401 was recovered from an abdominal wound swab, and no data were available on linezolid treatment of this patient. Isolates were initially tentatively identified as S. aureus by using the tube coagulase test, as described previously (45), and as cefoxitin and linezolid resistant by disk diffusion using European Committee on Antimicrobial Susceptibility Testing (EUCAST) methodologies and interpretive criteria (46, 47). Definitive identification of isolates as S. aureus was performed by DNA microarray profiling (see below). Plasmid-free novobiocin-resistant S. aureus strain XU21 was used as a plasmid recipient in filter-mating experiments (48). Isolates were stored at −80°C on Protect Bacterial Preservation System cryogenic beads in individual preserver vials (Technical Services Consultants Ltd., Heywood, United Kingdom).

### Investigation of isolates for the PhLOPS_A_ phenotype.

The two linezolid-resistant MRSA isolates (M12/0145 and M13/0401); their respective *cfr*-negative, plasmid-cured derivatives (M12/0145-C1 and M13/0401-C1); and the *cfr*-positive transconjugant derivative of XU21 (M12/0145/XU21-T1), generated following mating experiments between M12/0145 and the recipient strain XU21, were investigated for the PhLOPS_A_ phenotype. Chloramphenicol, clindamycin, and linezolid MICs were determined by using the Vitek 2 system (AST P580 panel, susceptibility tests for Gram-positive bacteria; bioMérieux, Basingstoke, Hampshire, United Kingdom) according to the manufacturer's instructions. Tiamulin MICs were determined by using Etest strips ranging from 0.002 mg/liter to 32 mg/liter (Liofilchem, Roseto degli Abruzzi, Italy). Virginiamycin M_1_ MICs were determined by broth microdilution (range, 1 mg/liter to 256 mg/liter) using Clinical and Laboratory Standards Institute (CLSI) methodologies and virginiamycin M_1_ powder (Sigma-Aldrich Ireland Ltd., Arklow, County Wicklow, Ireland) (49). The absence of the PhLOPS_A_ phenotype in plasmid-free S. aureus recipient strain XU21 was determined as described previously (20).

### Additional antimicrobial susceptibility testing.

The two linezolid-resistant MRSA parental isolates, their cured and transconjugant derivatives, and recipient strain XU21 also underwent antimicrobial susceptibility testing against a panel of 23 antimicrobial agents and heavy metals according to EUCAST methodologies (47), using previously described interpretive criteria and quality control strains (50). The 23 agents tested were amikacin, ampicillin, cadmium acetate, chloramphenicol, ciprofloxacin, erythromycin, ethidium bromide, fusidic acid, gentamicin, kanamycin, lincomycin, mercuric chloride, mupirocin, neomycin, phenyl mercuric acetate, rifampin, spectinomycin, streptomycin, sulfonamide, tetracycline, tobramycin, trimethoprim, and vancomycin.

### Genotyping.

The two linezolid-resistant MRSA isolates and their cured derivatives underwent *spa* typing. Genomic DNA for *spa* typing was extracted from each isolate/derivative by using enzymatic lysis and the DNeasy blood and tissue kit (Qiagen, Crawley, West Sussex, United Kingdom) according to the manufacturer's instructions. PCRs were performed by using GoTaq Flexi DNA polymerase (Promega Corporation, Madison, WI, USA), according to the manufacturer's instructions, using the primers and thermal cycling conditions described by the European Network of Laboratories for Sequence Based Typing of Microbial Pathogens (SeqNet) (http://www.seqnet.org/) and a G-storm GS1 thermocycler (Applied Biosystems, Foster City, CA). PCR products were visualized by conventional agarose gel electrophoresis and were purified by using the GenElute PCR cleanup kit (Sigma-Aldrich). Sequencing was performed commercially by Source Bioscience (Tramore, Waterford, Ireland), using an ABI 3730xl Sanger sequencing platform. Ridom StaphType version 1.3 software (Ridom GmBH, Würzburg, Germany) was used for *spa* sequence analysis and assignment of *spa* types (51). The two linezolid-resistant MRSA isolates also underwent pulsed-field gel electrophoresis (PFGE) using SmaI, as described previously (52).

The StaphyType DNA microarray kit (Alere Technologies, Jena, Germany) was used for confirmation of isolates as S. aureus, for assigning isolates and derivatives to MLST STs and/or CCs and SCC*mec* types, and for detecting antimicrobial resistance genes (including *cfr*) and virulence genes (53, 54). The DNA microarray procedures were performed according to the manufacturer's instructions, and the primers, probes, and protocols were described previously in detail (53, 54). Genomic DNA for use with the DNA microarray kit was extracted from isolates and derivatives by enzymatic lysis using the buffers and solutions provided with the StaphyType kit and the Qiagen DNeasy blood and tissue kit (Qiagen, Crawley, West Sussex, United Kingdom). DNA microarray profiling of plasmid-free S. aureus recipient strain XU21 was performed in a previous study (20).

### Plasmid analysis and whole-genome sequencing.

Plasmid curing and filter-mating conjugative transfer experiments were performed as described previously (48, 55, 56). The two linezolid-resistant parental MRSA isolates underwent whole-genome sequencing (WGS) in order to (i) determine the genetic organizations of *cfr* and its surrounding regions in these isolates and compare them to each other and to those described previously; (ii) determine the number of single-nucleotide variants (SNVs) between the two linezolid-resistant MRSA isolates; (iii) assign the two linezolid-resistant MRSA isolates to MLST STs, as the DNA microarray assigned these isolates only to MLST CCs; (iv) identify any possible linezolid resistance-associated ribosomal target site mutations in the *rplC* (L3), *rplD* (L4), and *rplV* (L22) genes in the two *cfr*-positive MRSA isolates; and (v) detect *optrA* and *cfr*(B). The 23S rRNA alleles were amplified by PCR as described previously (57), and sequencing reactions were performed by Source Bioscience.

For both isolates, WGS was performed by using a MiSeq desktop sequencer (Illumina, Essex, United Kingdom), and, for M13/0401 only, WGS was also performed by using a PacBio RS sequencing system (Pacific Biosciences, USA) with subsequent Hierarchal Genome Assembly Process (HGAP.3) analysis (The Genome Analysis Centre [TGAC], Norwich, United Kingdom), to confirm the genetic organization of the novel *cfr* plasmid identified. Genomic DNA for WGS was extracted from both isolates by using the Qiagen DNeasy blood and tissue kit. For the MiSeq analysis, WGS libraries were prepared by using Nextera XT library preparation reagents (Illumina). Reads generated by using the MiSeq system were checked for quality, trimmed, and assembled into contigs by using the Velvet *de novo* assembler, which is incorporated into SeqSphere version 2.3 software (Ridom). For PacBio WGS, genomic DNA was checked for quality and concentration according to TGAC guidelines. Contigs generated from both WGS methods were analyzed separately by using the BioNumerics Genome Analysis Tool (GAT) plug-in (version 7.5; Applied Maths, Sint-Martens-Latem, Belgium), the Artemis genome browser and annotation tool (58), and BLAST software (http://blast.ncbi.nlm.nih.gov/Blast.cgi). Open reading frames (ORFs) were predicted by using the BioNumerics annotation tool and BLAST software packages. ORFs were aligned with best-fitting matches in GenBank, and the locations of start and stop codons were checked for consistency and modified if required. Any gaps identified in the *cfr* region in the isolates were closed by PCR and sequencing using primers based on the surrounding contigs followed by amplimer sequencing at Source Bioscience. Data were analyzed and overlapping sequences were assembled by using BioNumerics. The genetic organization of the *cfr* region in each isolate was confirmed by using PCR and the primers listed in Table S1 in the supplemental material. For M12/0145, this was done for the Δ*tnpA-fexA* region encompassing *cfr* and not the entire *cfr*-carrying plasmid in this isolate due to its high similarity to a previously described *cfr* plasmid. For M13/0401, this was done for the entire plasmid, as it was distinct from those described previously.

MiSeq WGS data for M13/0401 were also resequenced against the *de novo* MiSeq assembly of isolate M12/0145 followed by alignment, and SNVs were identified and confirmed if they exhibited ≥40× coverage; i.e., each SNV was covered by at least 40 reads, thereby avoiding ambiguous SNVs and increasing the confidence in the SNV validity. All synonymous and nonsynonymous mutations were included. Insertions and deletions (indels) and repetitive regions were excluded.

### Nucleotide sequence accession numbers.

The nucleotide sequences from M12/0145 and M13/0401 have been deposited in GenBank under the following accession numbers: KU521355 and KU510528 for *cfr*-carrying plasmids in M12/0145 and M13/0401, respectively; KU510534 for allele 1, KU510535 for allele 2, KU510536 for allele 3, KU510537 for allele 4, KU510538 for allele 5, and KU510539 for allele 6 of the 23S rRNA V domain of M12/0145; KU510529 for allele 1, KU510530 for allele 2, KU510531 for allele 3, KU510532 for allele 4, and KU510533 for allele 5 of the 23S rRNA V domain of M13/0401; and KU510541 and KU510540 for *rplV* (L22) in M12/0145 and M13/0401, respectively.

## RESULTS

### Phenotypic and genotypic characteristics of linezolid-resistant MRSA.

Both isolates M12/0145 and M13/0401 were assigned to ST22-MRSA-IV and *spa* type t032. Each isolate exhibited the PhLOPS_A_ phenotype with linezolid MICs of 64 mg/liter (M12/0145) and 16 mg/liter (M13/0401) (Table 1). Both isolates lacked *optrA* and *cfr*(B) but harbored *cfr*, and one isolate (M12/0145) also harbored the phenicol exporter gene *fexA* (Table 1). The isolates differed by five bands in the PFGE analysis and 603 SNVs following WGS analysis (MiSeq coverages of 131× and 170× for M12/0145 and M13/0401, respectively). Both *cfr*-positive MRSA isolates also exhibited resistance to ampicillin, erythromycin, lincomycin, ciprofloxacin, and fusidic acid and carried the resistance genes *blaZ* and *erm*(C). Isolate M13/0401 was also resistant to rifampin (Table 1). Both isolates harbored the enterotoxin C gene *sec* and the enterotoxin gene cluster *egc* but differed by the presence of immune evasion complex (IEC) genes in isolate M12/0145 (Table 1).

**TABLE 1:** Phenotypic and genotypic characteristics of the parental linezolid-resistant ST22–MRSA-IV isolates and their cured and transconjugant derivatives

Isolate or derivative^*a*^	CC/ST-SCC*mec* type	*spa* type^*f*^	*cfr* and *fexA* carriage	Presence of PhLOPS_A_ phenotype^*c*^	PhLOPS_A_ agent MIC (mg/liter)^*c*^					Resistance to other antimicrobial agent(s)^*d*^	Other resistance genes^*e*^	Virulence gene(s)
					LZD	CHL	CLI	TIA	VIR			
M12/0145	CC/ST22-MRSA-IV	t032	*cfr* and *fexA*	Yes	64	128	2	>32	>256	AMP, CIP, ERM, FUC, LIN	*blaZ*, *erm*(C), *fexA*	*sec*, *egc*, IEC (*sak*, *chp*, and *scn*)
M12/0145-C1	CC/ST22-MRSA-IV	t032	None	No	8	0.25	0.5	1	8	AMP, CIP, ERM, FUC, LIN	*blaZ*, *erm*(C), *fexA*	*sec*, *egc*, IEC (*sak*, *chp*, and *scn*)
M12/0145/XU21-T1	CC8-MSSA	ND	*cfr* and *fexA*	Yes	8	>256	>256	>32	>256	None	*fosB*, *sdrM*	None
XU21^*b*^	CC8-MSSA	ND	None	No	1	8	0.25	1	1	None	*fosB*, *sdrM*	None
M13/0401	CC/ST22-MRSA-IV	t032	*cfr*	Yes	16	>256	>256	>32	>256	AMP, CIP, ERM, FUC, LIN, RIF	*blaZ*, *erm*(C) [*lsa*(B)]	*sec*, *egc*
M13/0401-C1	CC/ST22-MRSA-IV	t032	None	No	2	4	0.12	2	8	AMP, CIP, ERM, FUC, LIN, RIF	*blaZ*, *erm*(C)	*sec*, *egc*

a M12/0145 and M13/0401 are the *cfr*-positive parental isolates. Cured derivatives are indicated with “C1” after the parental isolate designations. The *cfr*- and *fexA*-positive transconjugant derivative M12/0145/XU21-T1 was generated by filter mating using M12/0145 as the plasmid donor and XU21 as the plasmid recipient. XU21 was the plasmid-free recipient strain used in conjugation experiments.

b The phenotypic and genotypic characteristics (apart from resistance to antimicrobial agents outside the PhLOPS_A_ phenotype) of plasmid-free S. aureus recipient strain XU21 were determined in a previous study (20).

c Resistance to phenicols (chloramphenicol [CHL]), lincosamides (clindamycin [CLI]), oxazolidinones (linezolid [LZD]), pleuromutilins (tiamulin [TIA]), and streptogramin A compounds (virginiamycin [VIR]) is indicative of the PhLOPS_A_ phenotype.

d The resistance of each isolate to the following antimicrobial agents was also determined: amikacin, ampicillin (AMP), cadmium acetate, ciprofloxacin (CIP), ethidium bromide, erythromycin (ERM), fusidic acid (FUC), gentamicin, kanamycin, lincomycin (LIN), mercuric chloride, mupirocin, neomycin, phenyl mercuric acetate, rifampin (RIF), sulfonamide, tetracycline, tobramycin, trimethoprim, and vancomycin.

e All resistance genes, apart from *lsa*(B), which is indicated in square brackets, were detected by DNA microarray profiling using the StaphyType kit (Alere). *lsa*(B) was detected in isolate M13/0401 in close proximity to *cfr* from the whole-genome sequence.

f ND, not determined.

### Characterization of the genetic environment of *cfr* in ST22-MRSA-IV isolates.

Whole-genome sequence analysis as well as results from plasmid-curing experiments indicated that *cfr* was plasmid located in both ST22-MRSA-IV isolates. *cfr*-positive isolate M13/0401 was successfully cured of *cfr*, whereas *cfr*- and *fexA*-positive isolate M12/0145 was successfully cured of both genes (Table 1). Cured derivatives of both isolates M12/0145-C1 and M13/0401-C1 lacked the PhLOPS_A_ phenotype but were otherwise indistinguishable from their respective parental isolates in terms of antimicrobial resistance phenotype, antimicrobial resistance and virulence genes detected by using DNA microarray analysis, and MLST SCC*mec* and *spa* types (Table 1). While the *cfr*-negative cured derivative M13/0401-C1 was linezolid susceptible, the *cfr*- and *fexA*-negative cured derivative M12/0145-C1 exhibited linezolid resistance, with a linezolid MIC of 8 mg/liter (Table 1), but this was lower than the corresponding linezolid MIC exhibited by its *cfr*-positive parental isolate (M12/0145 linezolid MIC of 64 mg/liter) (Table 1).

A transconjugant derivative of S. aureus recipient strain XU21 (M12/0145/XU21-T1) (Table 1) was obtained by using MRSA isolate M12/0145 as the donor; it exhibited the PhLOPS_A_ phenotype and was otherwise indistinguishable from XU21 apart from the presence of *cfr* and *fexA* (Table 1). Several separate attempts to generate a transconjugant derivative of XU21 using M13/0401 as the donor were unsuccessful. In contrast, isolates M05/0060 (a *cfr*-positive ST8-MRSA-IVa isolate and the only previously described *cfr*-positive MRSA isolate from Ireland) (20) and M12/0145 (*cfr*-positive ST22-MRSA-IV isolate) (this study), shown to harbor conjugative *cfr* plasmids, consistently yielded *cfr*-positive transconjugants when used as positive controls.

**FIG 1:**
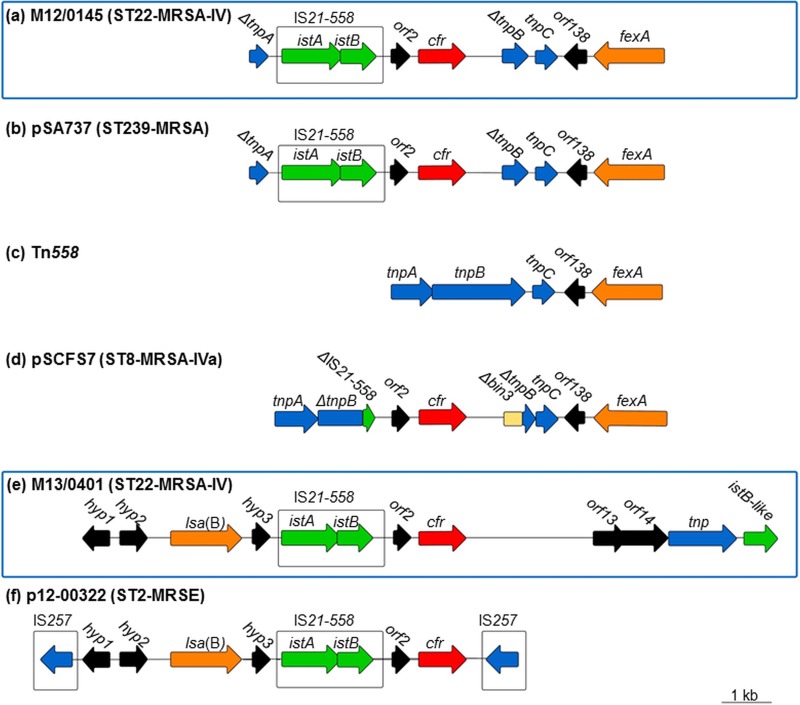
Schematic representation of the *cfr*-containing regions of ST22-MRSA-IV isolates M12/0145 (a) and M13/0401 (e) identified in the present study (surrounded by boxes [blue outline]) and previously described comparator plasmids and transposons, including pSA737 (GenBank accession no. KC206006) (29, 30) (b), Tn*558* (accession no. AJ715531) (4) (c), pSCFS7 (accession no. FN995111, FN995110, and FR675942) (20) (d), and p12-00322 (accession no. KM521836) (59) (f). Arrows indicate the direction of transcription of each ORF. Insertion sequence elements are surrounded by a box (black outline). Each gene or group of genes is represented by a different color; i.e., red indicates *cfr*, orange indicates antibiotic resistance genes other than *cfr*, green indicates IS*21-558* transposition genes, blue indicates other transposition genes, and black indicates genes (*hyp*) and ORFs encoding hypothetical proteins. Horizontal lines between ORFs indicate intergenic regions.

Based on the whole-genome sequence, the *cfr* plasmids in M12/0145 and M13/0401 were found to differ substantially from each other (Fig. 1a and e) and were identified on four and two contigs, respectively, following MiSeq WGS and, for M13/0401 only, on one contig following PacBio sequencing (PacBio coverage for M13/0401 of 100×). For isolate M12/0145, the *cfr*-carrying plasmid was 41,587 bp in size, and it was most similar in size and genetic organization to the previously reported 39-kb *cfr*-carrying plasmid pSA737 in an MRSA ST239 strain (GenBank accession no. KC206006; 94% DNA sequence homology). In fact, the genetic organization of the *cfr* region in M12/0145 was very similar to that described previously for pSA737/pSCFS3-like *cfr* plasmids from a diverse range of staphylococcal species from a variety of human and animal hosts (see Table S2 in the supplemental material). The region surrounding *cfr* in all of these plasmids, and in M12/0145 in the present study, consists of an IS*21*-like element (IS*21-558*) and *cfr* inserted into the *fexA*-carrying transposon Tn*558*, resulting in a truncation of the Tn*558* transposase genes *tnpA* and *tnpB* (Fig. 1a to d). The transposase genes Δ*tnpB* (Δ indicates a truncation) and *tnpC*, *orf138* (encoding a putative oxidoreductase), and *fexA* are located downstream of *cfr*, and *orf2*, IS*21-558* (consisting of two overlapping ORFs for *istA* and *istB*), and Δ*tnpA* are located upstream of *cfr* (Fig. 1a and b). The DNA sequences of the *cfr* region in M12/0145 and the *cfr* region in pSA737 differed only by a deletion of a thymine (T) nucleotide base in the intergenic region between *orf2* and *cfr* in M12/0145. However, beyond the *cfr* region, the only difference identified was a 2,326-bp region in M12/0145, located ca. 8 kb downstream of *cfr*, that is not present in pSA737. This region in M12/0145 consisted of a transposase gene and an *istB*-like gene with 48% DNA sequence homology to *istB* that may be involved in transposition.

The *cfr* region in M12/0145 was also compared to the corresponding region in ST8-MRSA-IVa isolate M05/0060 carrying pSCFS7, the only previously described MRSA isolate recovered in Ireland found to carry *cfr* (Fig. 1d). Although both *cfr* plasmids carried *fexA* and appeared to be derivatives of the insertion of IS*21-558* and *cfr* into Tn*558*, they differed mainly due to the insertion site of the IS element and *cfr* (Fig. 1a and d). In pSCFS7, the integration of the IS*21-558*–*cfr* region within Tn*558* resulted in a truncation of the IS element and *tnpB*, while in M12/0145, both *tnpA* and *tnpB* are truncated, but the IS*21-558* element is intact (Fig. 1a and d).

For isolate M13/0401, the *cfr*-carrying plasmid was 27,502 bp in size, and the region immediately upstream of *cfr* was similar to that in M12/0145 and consisted of *orf2* and IS*21-558* (Fig. 1a and e). The DNA sequences of these genes were 100% identical to those found in M12/0145. The *cfr* gene differed by one nucleotide base only, at position 983, between the two isolates (T in M12/0145 and G in M13/0401), resulting in different amino acids in M12/0145 (serine) and M13/0401 (arginine). In contrast to the *cfr* region in M12/0145, the ABC transporter gene *lsa*(B), encoding low-level lincosamide resistance, was also detected upstream of *cfr* in M13/0401 (Fig. 1e). This ABC transporter gene was previously detected in *cfr* plasmids p12-03322 (MRSE ST2) (Fig. 1f) (59), pSCFS6 (Staphylococcus warneri) (60), and pSCFS1 (Staphylococcus sciuri) (11). However, the latter two plasmids (pSCFS6 and pSCFS1) differ substantially from the *cfr*-containing region identified in M13/0401, with pSCFS6 also containing *fexA* and pSCFS1 harboring the spectinomycin resistance gene *spc* and the macrolide-lincosamide-streptogramin B resistance gene *erm* (33) but lacking IS*21-558*. The genetic organization of the *cfr* region in M13/0401 showed the highest overall similarity to that of p12-00322 (Fig. 1e and f). However, in p12-00322, the *cfr* region is flanked by IS*257* elements, which were not identified in M13/0401. Similar to M12/0145, the region downstream of *cfr* in M13/0401 contained a transposase gene and an *istB*-like gene with 48% DNA sequence homology to *istB*, but these were not identified in p12-00322 (Fig. 1e and f).

The remainder of the *cfr*-carrying plasmid in M13/0401 was also distinct from p12-00322. While 14 additional ORFs were identified in the *cfr* plasmid in M13/0401, it lacked the putative conjugation machinery (*tra*), encompassing the majority of the remainder of p12-00322 (59). A gene (*ssaA*) encoding an SsaA-like transposon-related protein was detected 5,648 bp upstream of *lsa*(B) in M13/0401. The *ssaA* gene exhibited 63.3% DNA sequence homology to *ssaA* present on Staphylococcus cohnii *cfr*-containing plasmid pHK01 (61) and 48.8% DNA sequence homology to *ssaA* on plasmids pSK73 (S. aureus) (GenBank accession no. GQ915269.1) and p12-02300 (MRSE ST2) (59). A BLAST search of the amino acid sequences of other predicted ORFs identified within the DNA sequence of the *cfr*-carrying plasmid in M13/0401 indicated that although the percentage of homology was low (30 to 40%), a number of these proteins exhibited amino acid identity to proteins involved in DNA transfer, including a variety of proteins from bacilli and staphylococci involved in conjugation (see Table S3 in the supplemental material). The remaining predicted ORFs exhibited similarity to hypothetical proteins only.

### Characterization of ribosomal mutations associated with linezolid resistance.

The same two mutations were detected in multiple 23S rRNA alleles and in L22 of both *cfr*-positive ST22-MRSA-IV isolates. These mutations included a change from guanine to thymine at nucleotide position 2603 (in 2/6 alleles in M12/0145 and in 1/5 alleles in M13/0401) in the V domain of the 23S rRNA gene and an amino acid change from alanine to valine at position 29 in L22. No amino acid changes were detected in the L3 or L4 proteins of either isolate.

## DISCUSSION

The ST22-MRSA-IV clone is a pandemic nosocomial MRSA clone, and previous studies have revealed the ability of this clone to adapt to the introduction of different antimicrobial agents into the health care environment (38). In the present study, we report another step in the evolution of this MRSA clone, with the first report of the transferable multidrug resistance gene *cfr* in two independent ST22-MRSA-IV isolates. Although both isolates were from patients in Irish hospitals, they were epidemiologically unrelated, i.e., from two geographically disparate hospitals. In addition, although both isolates were assigned to *spa* type t032, and only a single difference was detected in their antimicrobial resistance phenotypes (rifampin resistance in one isolate only), DNA microarray profiling revealed some differences in terms of an additional antimicrobial resistance gene (*fexA*) and virulence gene complex (IEC) in one isolate. Whole-genome sequence analysis ultimately provided the definitive evidence that these two ST22-MRSA-IV isolates were genotypically as well as epidemiologically distinct, due to the large numbers of SNVs identified (603 SNVs).

Detailed plasmid analysis of the two ST22-MRSA-IV isolates revealed that *cfr* has been introduced on two distinct plasmids into ST22-MRSA-IV isolates. In MRSA ST22 isolate M12/0145, *cfr* and *fexA* were colocated on a conjugative plasmid that was very similar to pSA737 (29, 30), previously described in isolates of other MRSA genotypes and in a variety of coagulase-negative staphylococcal (CoNS) species from both animals and humans (see Table S3 in the supplemental material). Plasmid pSA737 is a pSCFS3-type plasmid, one of the most common types of *cfr*-containing plasmids. While the genetic environment of *cfr* in the second ST22-MRSA-IV isolate (M13/0401) revealed some similarities to that in M12/0145 in terms of the presence and location of *orf2* and the IS*21-558* transposase genes *istAS* and *istBS*, it was otherwise distinct from the plasmid in M12/0145. In fact, the *cfr* region in M13/0401 showed the most similarity to that in MRSE plasmid p12-00322, with both harboring *lsa*(B), but both *cfr* regions were carried on otherwise distinct plasmids. Genes with homology to those involved in mobility were identified in M13/0401, but the *tra* genes of p12-00322 were absent. Despite repeated attempts, filter-mating experiments using M13/0401 as a donor failed to yield any transconjugants, suggesting that the *cfr*-carrying plasmid present in M13/0401 was nonconjugative, at least under the conditions tested.

Anecdotal data on two additional linezolid-resistant ST22-MRSA-IV isolates recovered from two other patients in the same hospital as the one where M13/0401 was recovered, and within 3 months of the isolation of M13/0401, indicated that these two isolates were indistinguishable from M13/0401 based on antimicrobial susceptibility testing, *spa* typing, and DNA microarray data (data not shown). Although these two isolates were originally phenotypically linezolid resistant and *cfr* positive by PCR, they were subsequently found to be linezolid susceptible and to lack *cfr* following storage and subculturing, indicating the instability of the *cfr*-carrying plasmid in these isolates. However, the recovery of three genotypically indistinguishable *cfr*-positive isolates from patients in the same hospital in a similar time frame suggests the ability of this *cfr*-positive ST22-MRSA-IV strain to spread between patients. The patient from whom M12/0145 was recovered was also found to harbor an indistinguishable ST22-MRSA-IV strain based on DNA microarray profiling and *spa* typing that was linezolid susceptible and lacked *cfr* and the PhLOPS_A_ phenotype (data not shown). Furthermore, the patient from whom M12/0145 was recovered had been treated previously with linezolid. This isolate may represent a precursor to the *cfr*-positive ST22-MRSA-IV isolate identified in the present study or an example of the loss of *cfr* in this strain.

The origin of the *cfr*-carrying plasmids in these ST22-MRSA-IV isolates is as yet unknown. Both plasmids were distinct from a previously reported *cfr*-carrying plasmid from a ST8-MRSA-IV isolate characterized in Ireland (20). The *cfr*-carrying plasmid in M12/0145 may have spread from other staphylococci, either S. aureus or CoNS, as the same plasmid types have been reported previously, in both human and animal staphylococcal isolates. The *cfr*-carrying plasmid from M13/0401 is distinct from those described previously, but similarities to those in MRSE suggest CoNS as a possible source. Recent reports of *cfr*-harboring MRSE isolates in Ireland raise the possibility that MRSE may be the source of these *cfr* plasmids, although analysis of the *cfr* region in these MRSE isolates has not yet been reported, so a comparison is not possible (34, 35). Enterococci could also be the source of *cfr* in the ST22-MRSA-IV isolates, as linezolid resistance appears to be more common among enterococci. Only a single *cfr*-positive linezolid-resistant enterococcal isolate has been reported from Ireland, with no detailed plasmid analysis (62). Detailed systematic analysis of additional staphylococcal and enterococcal isolates from both animals and humans in Ireland for *cfr* is necessary to determine the source of these *cfr* plasmids and to prevent further spread.

Both *cfr*-positive ST22-MRSA-IV isolates also harbored a mutation in 23S rRNA (G2603T), and this mutation was shown previously to confer linezolid resistance in S. aureus and S. epidermidis (32, 63). Isolate M12/0145 exhibited a linezolid MIC of 64 mg/liter and harbored mutations in two 23S rRNA alleles, while isolate M13/0401 exhibited a linezolid MIC of 16 mg/liter and harbored mutations in one 23S rRNA allele, suggesting a possible relationship between the number of mutated alleles and the linezolid MIC. Furthermore, while curing both isolates of their *cfr*-carrying plasmids resulted in a reduction in their respective linezolid MICs, the cured derivative of M13/0401 was linezolid susceptible (linezolid MIC of 2 mg/liter), while that of M12/0145 (which had the two mutated 23S rRNA alleles) remained borderline linezolid resistant (linezolid MIC of 8 mg/liter). Mutations were also detected in the gene for the L22 protein, which resulted in the amino acid substitution A29V in both isolates. Little is known about the effects, if any, of L22 mutations on linezolid resistance, although it is assumed that L22 plays a role due to its close proximity to the linezolid binding site (64). The presence of distinct *cfr*-carrying plasmids in two ST22-MRSA-IV isolates indicates independent acquisition, and this, combined with mutation-mediated linezolid resistance, suggests that exposure to linezolid may have played a role in their emergence. Alternatively, since *cfr* encodes resistance to multiple antimicrobial agents, and because of the colocation of *cfr* on plasmids with other resistance genes in these isolates, i.e., *fexA* and *lsa*(B), other antimicrobial agents may provide the selective pressure for the emergence of *cfr*.

The identification of *cfr* in two distinct ST22-MRSA-IV strains is alarming. The distinct plasmids identified highlight the ability of *cfr* to spread and to complicate treatment options. Prudent management of linezolid usage is essential to prevent linezolid resistance from becoming more widespread.

## Supplementary Material

Supplemental material
