# Supplementary material for: First Report of cfr-Carrying Plasmids in the Pandemic Sequence Type 22 Methicillin-Resistant Staphylococcus aureus Staphylococcal Cassette Chromosome mec Type IV Clone
Source: Antimicrob Agents Chemother. 2016 Apr 22;60(5):3007–15. doi: 10.1128/AAC.02949-15 (PMC4862533; doi:10.1128/AAC.02949-15)
Supplement: Supplemental material [file supp_60_5_3007__index.html]

Supplemental material 

# First Report of *cfr*-Carrying Plasmids in the Pandemic Sequence Type 22 Methicillin-Resistant Staphylococcus aureus Staphylococcal Cassette Chromosome *mec* Type IV Clone

## Supplemental material

- Supplemental file 1 -

  Supplemental Tables S1 to S3

  PDF, 259K
