# Supplementary material for: First Report of cfr-Carrying Plasmids in the Pandemic Sequence Type 22 Methicillin-Resistant Staphylococcus aureus Staphylococcal Cassette Chromosome mec Type IV Clone
Source: Antimicrob Agents Chemother. 2016 Apr 22;60(5):3007–15. doi: 10.1128/AAC.02949-15 (PMC4862533; doi:10.1128/AAC.02949-15)
Supplement: Supplemental material [file AAC.02949-15_zac005165157so1.pdf]

Supplemental Table S1. Primers used to confirm the genetic organization and orientation of the *cfr* region in the ST22-MRSA-IV isolates M12/0145 and M13/0401

Isolate no.	Gene/region amplified	Primer name	Nucleotide sequence (5'-3')	Nucleotide coordinates[a]	Amplimer size (bp)
M12/0145	*ΔtnpA - cfr*	tnpAF	GGTTCAGAAAGTAATTGTGGAGGA	31961-31984	4400
		cfrR	CCTATAATTGACCACAAGC	36343- 36361	
	cfr - tnpC	cfrF	GACTTTCGGCACCGGTAAT	35243-35261	2887
		tnpCR	GTTCATTCTCTTCTTCTAAGGCCTT	38106-38130	
	tnpC - fexA	tnpCF	CAGCTAGCTAAAGACAAGTCGGA	37842-37864	2781
		fexAR	GAGAACCGAATCTTTAATCA	40604-40623	
M13/0401	*orf1 – orf7*	orf1F	CAGTCATAGGCACACAAAC	776-794	8350
		orf7R	GCAACCAGTCAACAAGATC	9108-9126	
	ssaA - intergenic region between *ssaA* & *hyp1*	ssaAF	GGTAACTATGACAGACGGTTATAGC	9013-9037	3098
		intssaAR	GCTATATTGTGGCTC TGC	12096-12111	
	Intergenic region between *ssaA* & *hyp1* - *hyp1*	intssaAF	GGACAATTGCCATTAACG	11813-11830	3155
		0401hyp1R	CCTTTTGCATATCCCTAC	14951-14968	
	hyp1 – intergenic region between *hyp* and *istA*	HypF	CCAGCTGTTTAATTGGTTG	14801-14819	2530
		IntR	CGATATATTTGGATACGTG	17313-17331	

Intergenic region between *hyp* and *istA* - *istB*	BPF	GGAAAACGAGGAGTGATTACG	17205-17225	2238
	istBSR	CGATTTATGCGTCAAGC	19427-19443	
istB - *cfr*	istBSF	CCTCAACCATTATTACGAGC	19340-19359	1746
	cfrR	CCTATAATTGACCACAAGC	20168-21086	
cfr – o*rf1*	cfrF1	GACTTTCGGCACCGGTAAT	19967-19985	8539
	CR2	CCTTTATTCGCTCTTACATCACG	982-1004	

[a]Nucleotide coordinates based on the nucleotide sequence of the *cfr* region (*ΔtnpA – fexA*) in M12/0145 and the entire plasmid in M13/0401 (Genbank accession no. X and X, respectively; accession numbers pending).

Supplemental Table S2. *cfr*-containing pSA737- and pSCFS3-type plasmids previously identified in staphylococci from animals and humans showing a similar genetic organization to the *cfr* region identified in M12/0145 in the present study[a]

Plasmid name	Region sequenced	Nucleotide sequence identity to other *cfr* regions	Staphylococcal species	Genotype (*n*)[a]	Host	Year of isolation	Country of origin	Genbank accession no.	Reference
pSA737	Entire plasmid	99.7% to pSCFS3	MRSA	ST239-t037	Human clinical	2007	USA	KC206006	(1, 2)
p2823634	5.5 kb IS21-558 to **Δ***tnpB*	100% to pSA737	MRSA	USA300	Human clinical	2011	USA	KJ819951	(3)
p2823586	5.5 kb IS21-558 to **Δ***tnpB*	100% to pSA737	MRSA	USA300	Human clinical	2011	USA	KJ819952	(3)
p2823605	5.5 kb IS21-558 to **Δ***tnpB*	100% to pSA737	MRSA	USA300	Human clinical	2011	USA	KJ819953	(3)
pSCFS3	9.5 kb **Δ***tnpA* to *fexA*	99.7% to pSA737	*Staphylococcus aureus*	NA	Porcine respiratory tract infection	2000	Germany	AM086211	(4)
pSCFS3-type	*cfr*-containing BglII fragments	ND (similar *cfr* region to pSCFS3)	MRSA	ST398-t034	Porcine nares	2007	Germany	NA	(5)
pSCFS3-type	*cfr*-containing BglII fragments	ND (similar *cfr* region to pSCFS3)	MSSA	ST9-t3198	Porcine nares	2007	Germany	NA	(5)
pSEPI8573/pSE1243	Entire plasmid	100% to pSA737	MRSE	ND	Human clinical	2008-'09	USA	KC222021	(2)
pHNTLD18	5.7 kb EcoRI *cfr* fragment	100% to pSA737	*Staphylococcus equorum*	NA	Retail meat	2012	China	KF751702	(6)
pSS-02	14 kb *cfr* region	99.8% to pSCFS3	*Staphylococcus saprophyticus* & *Staphylococcus sciuri*	NA	Porcine nares	2010	China	JF834910	(7)
pSS-02-type	14 kb *cfr* region	100% to pSS-02	*Staphylooccus haemolyticus* & *Staphylococcis cohnii*	NA	Human clinical blood culture	2009-'10	China	JX827253	(8)

pHNCR35	10 kb *radC* to *fexA*	ND	*Staphylococcus simulans*	NA	Human hog market worker	NA	China	KF861983	Unpublished Genbank accession no. KF861983.1
pSS-02-type	*cfr* flanking regions	ND (similar *cfr* region to pSCFS3)	MRSA	ST627-t002-dt12w-IVb (3); ST6-t304-dt12w-IVb (2); ST63-MRSA-t899-dt12v-IVb (1)	Porcine nares & lungs	2012/13	China	NA	(9)

[a]The genetic organization of pSA737- and pSCFS3-type plasmids consists of **Δ***tnpA*-IS21-558 (*istAS* & *isaBS*)-*cfr*-**Δ***tnpB*-*tnpC*-*orf138*-*fexA*.
[b]Where available, multilocus sequence types, *spa* types and *dru* types are indicated with the prefixes ST, t and dt, respectively. USA300 genotype was determined by pulsed-field gel electrophoresis. Where available SCC*mec* types are indicated with roman numerals and subtypes with alphabetic designations. *n*, number of isolates and is only indicated where more than one isolate was identified.
NA, not applicable; ND, not determined.

REFERENCES

1. **Mendes RE, Deshpande LM, Castanheira M, DiPersio J, Saubolle MA, Jones RN.** 2008. First report of *cfr*-mediated resistance to linezolid in human staphylococcal clinical isolates recovered in the United States. Antimicrob Agents Chemother **52:**2244-2246.

2. **Mendes RE, Deshpande LM, Bonilla HF, Schwarz S, Huband MD, Jones RN, Quinn JP.** 2013. Dissemination of a pSCFS3-like *cfr*-carrying plasmid in *Staphylococcus aureus* and *Staphylococcus epidermidis* clinical isolates recovered from hospitals in Ohio. Antimicrob Agents Chemother **57:**2923-2928.

3. **Locke JB, Zuill DE, Scharn CR, Deane J, Sahm DF, Goering RV, Jenkins SG, Shaw KJ.** 2014. Identification and characterization of linezolid-resistant *cfr*-positive *Staphylococcus aureus* USA300 isolates from a New York City medical center. Antimicrob Agents Chemother **58:**6949-6952.

4. **Kehrenberg C, Schwarz S.** 2006. Distribution of florfenicol resistance genes *fexA* and *cfr* among chloramphenicol-resistant *Staphylococcus* isolates. Antimicrob Agents Chemother **50:**1156-1163.

5. **Kehrenberg C, Cuny C, Strommenger B, Schwarz S, Witte W.** 2009. Methicillin-resistant and -susceptible *Staphylococcus aureus* strains of clonal lineages ST398 and ST9 from swine carry the multidrug resistance gene *cfr*. Antimicrob Agents Chemother **53:**779-781.

6. **Zeng ZL, Wei HK, Wang J, Lin DC, Liu XQ, Liu JH.** 2014. High prevalence of Cfr-producing *Staphylococcus* species in retail meat in Guangzhou, China. BMC Microbiol **14:**151.

7. **Wang Y, He T, Schwarz S, Zhou D, Shen Z, Wu C, Wang Y, Ma L, Zhang Q, Shen J.** 2012. Detection of the staphylococcal multiresistance gene *cfr* in *Escherichia coli* of domestic-animal origin. J Antimicrob Chemother **67:**1094-1098.

8. **Cui L, Wang Y, Li Y, He T, Schwarz S, Ding Y, Shen J, Lv Y.** 2013. Cfr-mediated linezolid-resistance among methicillin-resistant coagulase-negative staphylococci from infections of humans. PLoS One **8:**e57096.

9. **Li D, Wu C, Wang Y, Fan R, Schwarz S, Zhang S.** 2015. Identification of Multiresistance Gene c*fr* in Methicillin-Resistant *Staphylococcus aureus* from Pigs: Plasmid Location and Integration into a Staphylococcal Cassette Chromosome *mec* Complex. Antimicrob Agents Chemother **59:**3641-3644.

Supplemental Table S3. Details of predicted open reading frames (ORFs) identified within the *cfr* plasmid of M13/0145 exhibiting amino acid identity to proteins involved in horizontal gene transfer

ORFs	Closest similiarity (Genbank accession no.)	% amino acid identity (query coverage)	Conserved protein domain family	Function
1	TraG bacilli(WP_021038 275.1)	38% (98%)	SXT_TraD	Conjugal transfer protein
2B	SAPIG1862 staphylococci (WP_031882362.1)	32% (87%)	TcpC	Conjugative transposon protein
3	pGIAK1_5 bacilli (AGQ45426.1)	37% (96%)	TcpE	Putative conjugative transposon membrane protein
4	VirB4 family protein bacilli (WP_021038260.1)	39% (97%)	MYSc_Myo14	Conjugal transfer and type IV secretion systems
10	Ssb *Staphylococcus aureus* (WP_012818034.1)	36% (84%)	ssb	Binding of single stranded DNA
